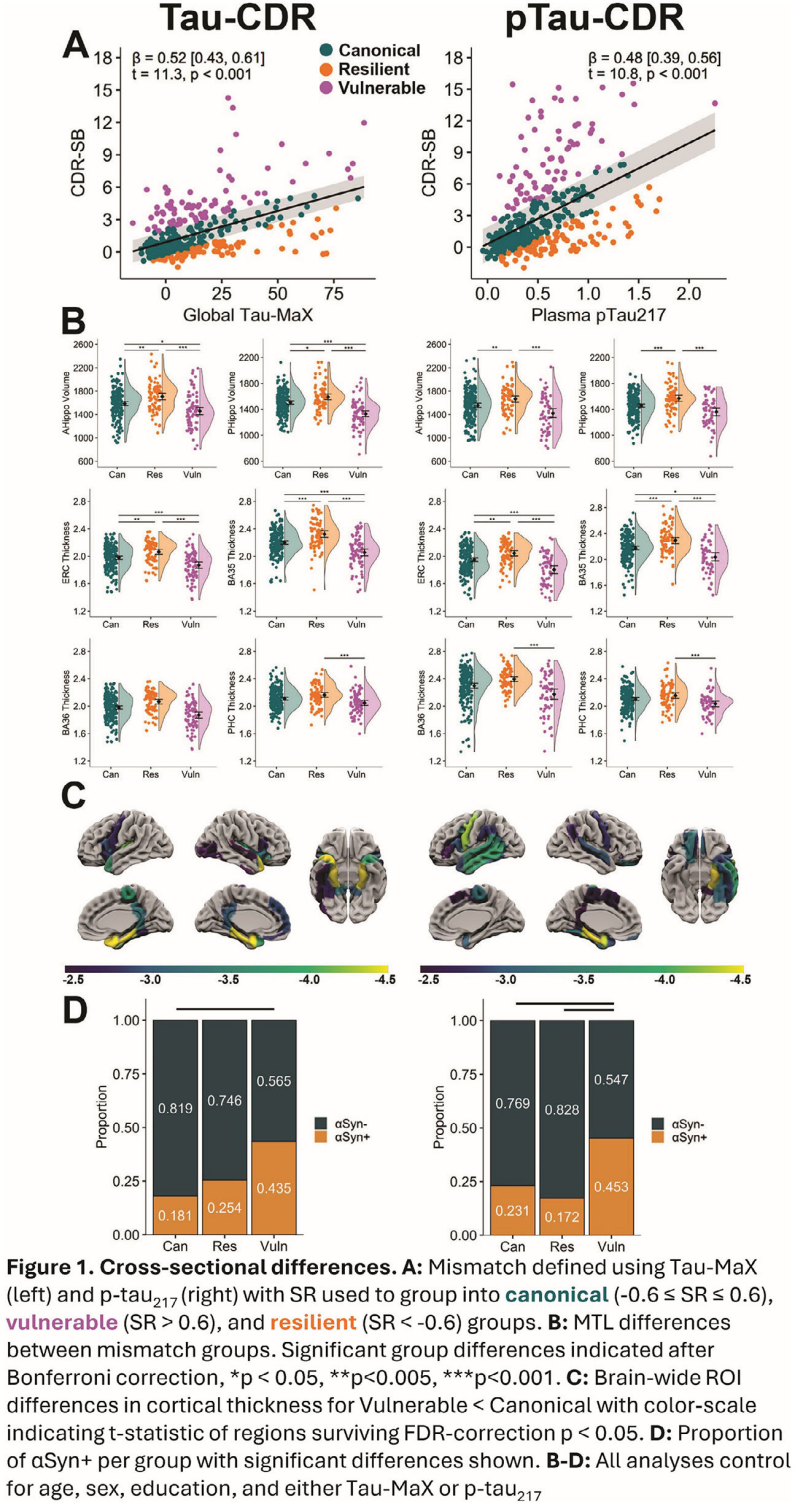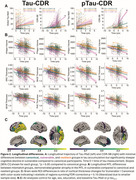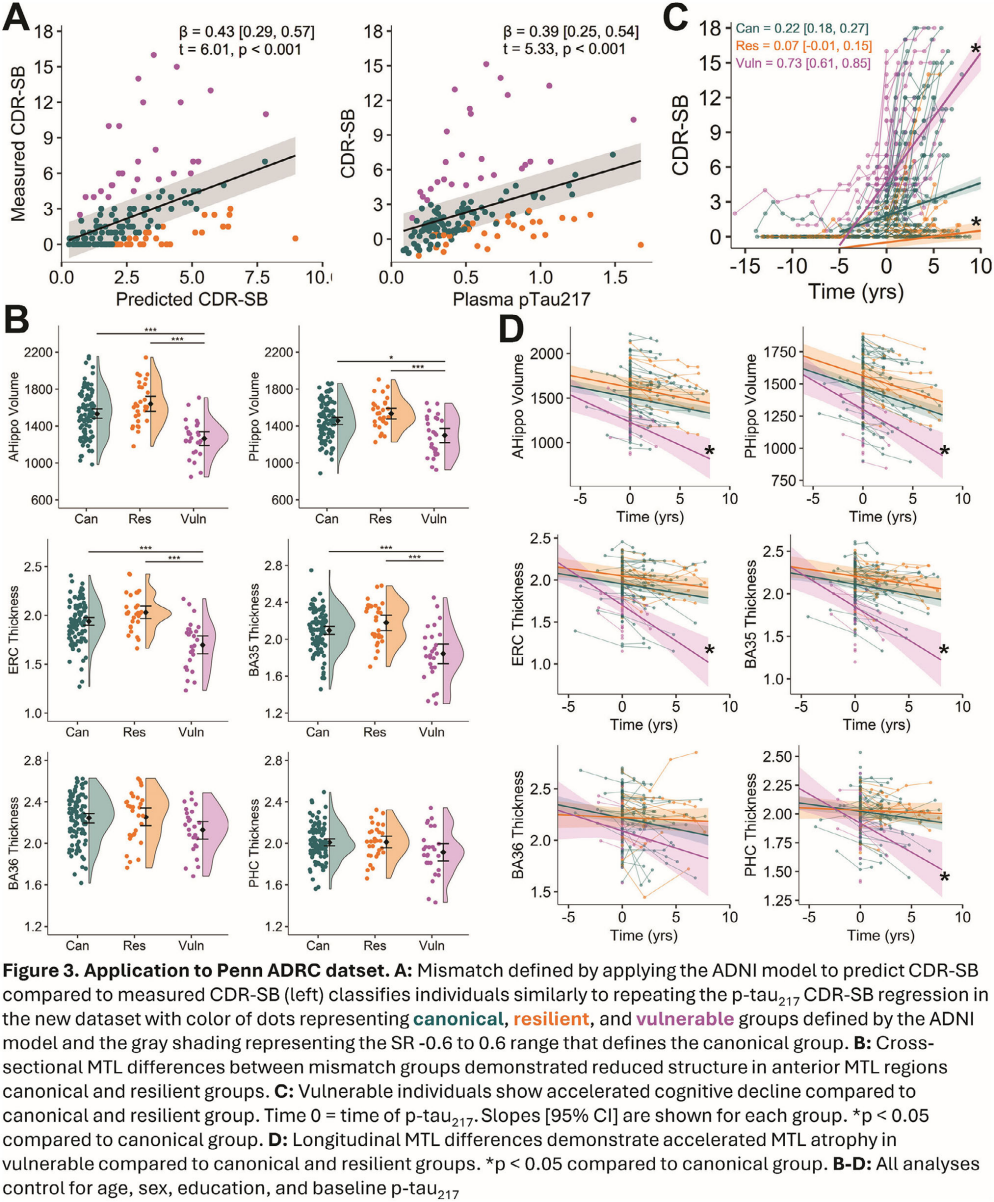# Tau Cognitive Mismatch in Alzheimer's Disease Predicts Presence of Co‐Pathology and Diverging Disease Trajectory

**DOI:** 10.1002/alz70856_104485

**Published:** 2025-12-26

**Authors:** Christopher A Brown, Sandhitsu R. Das, Katheryn A. Q. Cousins, Long Xie, Emily McGrew, Magdalena Korecka, Thomas F. Tropea, Alice Chen‐Plotkin, John A. Detre, Corey T. McMillan, Eddie B Lee, Ilya M Nasrallah, Paul A. Yushkevich, Dawn Mechanic‐Hamilton, Leslie M. Shaw, David A. Wolk

**Affiliations:** ^1^ University of Pennsylvania, Philadelphia, PA, USA; ^2^ Siemens Healthineers, Princeton, NJ, USA; ^3^ Perelman School of Medicine, University of Pennsylvania, Department of Pathology and Laboratory Medicine, Philadelphia, PA, USA

## Abstract

**Background:**

The revised Alzheimer's disease staging criteria use the concept of biological‐clinical mismatch with an expected, canonical progression; identifying individuals who have greater clinical impairment than expected for their biological stage as vulnerable or less impairment than expected as resilient. Here we used T1 (plasma *p*‐tau_217_) and T2 (^18^F‐flortaucipir PET) biomarkers to identify mismatch with global clinical severity measured by the CDR‐SB scale. We then investigated differences in brain structure, alpha synuclein positivity, and longitudinal trajectories of cognition, tau accumulation, and atrophy between canonical, vulnerable and resilient groups.

**Methods:**

Amyloid positive ADNI participants with available Tau PET (*n* = 373) or *p*‐tau_217_ (*n* = 392) and CDR‐SB score were included. Mismatch was defined using linear regression between global measures of tau burden, using a composite score of PET binding intensity and extent (Tau‐MaX) or *p*‐tau_217_, and CDR‐SB to divide individuals into canonical, vulnerable, and resilient groups. We investigated differences in medial temporal lobe (MTL) structure measured using T1‐ASHS, brain‐wide thickness differences using ANTs, and α‐synuclein status measured with CSF seed amplification assay after controlling for age, sex, education, and global tau burden. We examined longitudinal tau PET, atrophy, and cognitive trajectories between mismatch groups using linear mixed models controlling for age, sex, education, and global tau burden. Finally, we applied the mismatch model generated in ADNI to our local Penn ADRC dataset (*n* = 160) for replication.

**Results:**

Both Tau‐MaX and *p*‐tau_217_ were moderately associated with CDR‐SB (Figure 1A). Vulnerable individuals had greater neurodegeneration in anterior MTL and temporolimbic regions compared to canonical and resilient groups (Figure 1B‐C), as well as higher prevalence of α‐synuclein co‐pathology (Figure 1D). The vulnerable group showed accelerated cognitive decline even after controlling for rate of tau accumulation, which minimally differed by group (Figure 2A). Vulnerable participants also had accelerated atrophy in MTL and temporolimbic regions (Figure 2B‐C). Results in the Penn ADRC dataset (Figure 3A) were consistent with the ADNI dataset findings (Figure 3B‐D).

**Conclusions:**

Tau‐cognitive mismatch identifies vulnerable individuals who are more likely to have α‐synuclein co‐pathology and neurodegeneration patterns that overlap with LATE. The plasma‐based models also successfully identified similar vulnerable individuals in an independent dataset.